# Enhanced Immune Protection of Mud Crab *Scylla paramamosain* in Response to the Secondary Challenge by *Vibrio parahaemolyticus*

**DOI:** 10.3389/fimmu.2020.565958

**Published:** 2020-10-20

**Authors:** Xin Zhang, Xinyang Zeng, Yulong Sun, Yilei Wang, Ziping Zhang

**Affiliations:** ^1^College of Animal Science, Fujian Agriculture and Forestry University, Fuzhou, China; ^2^Fujian Engineering Research Center of Aquatic Breeding and Healthy Aquaculture, Jimei University, Xiamen, China; ^3^Key Laboratory of Healthy Mariculture for the East China Sea, Ministry of Agriculture, Fisheries College, Jimei University, Xiamen, China; ^4^Key Laboratory of Marine Biotechnology of Fujian Province, College of Animal Science, Institute of Oceanology, Fujian Agriculture and Forestry University, Fuzhou, China

**Keywords:** antimicrobial peptides, toll-like receptor signaling pathway, *Scylla paramamosain*, immune priming, gene expression

## Abstract

“Immune priming” plays a vital part in the immune system of invertebrates, protecting against recurrent infections by pathogens, and can provide some ideas for the prevention and treatment of invertebrate diseases. Many invertebrates have been demonstrated recently to have immune priming, but the relevant mechanisms are not known. Expression of immune system–related genes in the hemocytes and hepatopancreas of the mud crab (*Scylla paramamosain*) before and after repeated stimulation with *Vibrio parahaemolyticus* were analyzed by real-time fluorescence quantitative polymerase chain reaction. Some molecules that may participate in the immune priming of *S. paramamosain* were screened out, and their possible roles in immune priming were interpreted. Crabs injected first with heat-killed *V. parahaemolyticus* (HkVp group) or physiologic (0.9%) saline (PS group) were rechallenged at 168 h with live *V. parahaemolyticus* (HkVp+Vp group and PS+Vp group, respectively). The log-rank test shows a significant difference in survival rate between the HkVp+Vp group and the other groups after the ICH (*p* < 0.05). Expression of genes involved in the toll-like receptor (TLR) signaling pathway and some antimicrobial peptide genes were detected. By, respectively, comparing gene quantification at different time points in hemocytes and the hepatopancreas, the molecules that may play a part in the early stage of the immune priming of *S. paramamosain* in the hemocytes are found to be down syndrome cell adhesion molecule (Dscam), Hyastatin, Cactus, Arasin, antilipopolysaccharide factor 3 (ALF3), ALF4, ALF5, and ALF6 as well as later acting molecules, such as Crustin, Dorsal, Pelle, and myeloid differentiation factor 88 (MyD88). The molecules that functioned throughout the entire period are TLR and Spaetzle. In the hepatopancreas, the molecules that may play a part in the early stages of immune priming are Dscam, Hyastatin, Arasin, ALF6, Pelle, Spaetzle, Dorsal and, in the later stage, ALF4. The molecules that functioned throughout the entire period are TLR, Crustin, Cactus, MyD88, ALF3, and ALF5. In summary, the immune function of *S. paramamosain* is enhanced after it receives the same repetitive stimulation by *V. parahaemolyticus*, indicating immune priming in *S. paramamosain*. Our study enriches research on immune priming in invertebrates and lays the foundation for further studies revealing the molecular mechanism of immune priming in crabs.

## Introduction

Since the clonal-selection and gene-rearrangement theories were first proposed, significant progress has been achieved in the research of adaptive immunity (1, 2). It is considered that invertebrates lack the “true” memory B/T lymphocytes and antigen-specific immunoglobulin (Ig) molecules, which are the prerequisites and most striking features of the adaptive immune system of vertebrates (3, 4). Hence, it is a popular view that there is no adaptive immune response in invertebrates (5). Invertebrates can survive infection from various environmental pathogens and continue to have offspring, mainly by relying on their complex innate immune system.

The immune system of invertebrates has been considered to be nonspecific. However, increasing numbers of studies show that, if invertebrates are infected repeatedly by pathogenic microorganisms, a phenomenon similar to the immune memory found in vertebrates is observed (3, 4, 6–8), but the priming effect is not universal across all bacterial strains (9). The improved survival and immune responses observed after secondary exposure to a pathogen encountered previously in invertebrates is defined as “the ability to store and recall information on previously encountered microbial communities or their components” (10) and is called “immune priming” (11), “specific immune priming” (12), or “quasi-immune response” (13).

Initially, the immune memory of invertebrates was found only in starfish (*Dermasterias imbricata*), ribbon worm (*Lineus sanguineus*), earthworm (*Lymbricus terrestris*), and cockroach (*Periplaneta americana*) (14–17). However, often the results were controversial because the genotypes of animals used in the experiments could not be determined. This problem hindered the progress of research on the immune memory of invertebrates at that time.

Later, the study of the immune memory of invertebrates focused on the interaction between the host and parasites/bacteria. It was found that the immune memory of *Macrocyclops albidus* could be induced by *Schistocephalus solidus* (18). Since then, similar experimental conclusions have been reported in *Bombus terrestris* and *Paenibacillus species*, *Daphnia magna*, and *Pasteuria ramosa* (19, 20). Subsequently, some researchers questioned if this type of immune priming was overly dependent on the experimental conditions (21, 22). They note that most studies evaluate only the survival rate and fecundity of the host instead of measuring the expression of immune system–related genes or the infection rate of parasites/pathogens (8, 23, 24).

In recent years, “omics” research in invertebrates has revealed that some highly variable receptor molecules might be involved in the specific immune priming of invertebrates: scavenger receptor cysteine-rich proteins and immune-response proteins (25–27), down syndrome cell adhesion molecule (Dscam) (28–31), and fibrinogen-related proteins (FREPs) (32–34). In particular, the FREPs of *Biomphalaria glabrata* have been shown to interact with the mucin (SmPoMucs) of *Schistosoma mansoni* to form an immune complex, which is important evidence of the interaction between diverse immune receptors and antigen variation in the invertebrate host/pathogen (35). In 2015, Coustau and colleagues showed that the interaction between *B. glabrata* and *S. mansoni* is an extremely complex immune process in which a large number of antigens, immune receptors, effectors, and antieffector systems are involved (36).

Dubief and colleagues demonstrate that the hemolymph phagocytosis of *Haliotis tuberculata* is enhanced after reinfection by *Vibrio harveyi* (37). De Melo and colleagues demonstrate that secondary infection by *Schistosoma japonicum* caused a significant increase in the number of granulocytes in hemolymph in *B. glabrata* and *Biomphalaria straminea* (38). Research into *Eriocheir sinensis* indicates that the survival rate of individuals immunized with inactivated *Aeromonas hydrophila* for 7 days was significantly higher than that of untreated individuals when they were infected with live *A. hydrophila*. The phagocytosis of hemocytes in the inactivated *A. hydrophila*-immunized group was significantly greater than that in the *Micrococcus luteus*-immunized group and could be maintained for a long time compared with that after the first immunization with *A. hydrophila*. The serum of the *A. hydrophila*–immunized group had stronger antibacterial activity against the same bacteria than against different bacteria and showed a certain specificity (39). Moreover, research into oysters shows that the toll-like receptor (TLR) pathway participated in enhanced immune protection for hemocytes defending against second stimulation by *Vibrio splendidus* (40). Therefore, enhanced immune protection is a universal phenomenon shared by all species and not provided exclusively by adaptive immunity in vertebrates. Also, Ig molecules or lymphocytes might not be the essential prerequisites for enhanced immune protection in all organisms.

The mud crab, *Scylla paramamosain*, is distributed widely throughout the Indo-Pacific region. It is found commonly in estuaries and mangrove areas. It has also been cultured along the southeast coasts of China. Recently, mass mortality occurred due to an outbreak of diseases caused by viruses, bacteria, and parasites (41). *V. parahaemolyticus* is a primary bacterial pathogen of *S. paramamosain*. It has been reported to cause “milky disease” with high mortality and has led to major losses in the mud-crab aquaculture industry (42–44). However, due to a lack of research into disease prevention, the culture of *S. paramamosain* has been hampered by infection by various pathogens, which has restricted the development of the breeding industry.

During disease control/prevention, the use of antibiotics and immune enhancers can cause an excessive immune response and environmental pollution, so it does not constitute an ideal strategy. Immune priming, if present in *S. paramamosain*, could be an essential defense against *V. parahaemolyticus*. Antimicrobial peptides (AMPs) have been reported to be important components of the host immune defense system against pathogen infection. In crab species, even in *S. paramamosain*, several AMPs have been discovered and characterized (45–47). In addition to AMPs, classical immune-related processes, such as the TLR signaling pathway, are also reported to be involved in the immunity of *S. paramamosain* against Gram-negative and -positive bacteria (48–50). Taken together, these observations indicate that AMPs and the TLR signaling pathway have vital roles in recognizing and eliminating *V. parahaemolyticus* during immune protection of *S. paramamosain*, which may also induce enhanced immune protection.

Hence, the main objectives of the present study were to (i) examine the different experimental treatments of *S. paramamosain* resistant to *V. parahaemolyticus* infection, (ii) compare the immune responses of different tissues (hemocytes and the hepatopancreas) during successive exposures to the pathogen and thereby explain differences in tolerance to the disease, and (iii) investigate if AMPs and the TLR signaling pathway take part in the immune priming of *S. paramamosain* against *V. parahaemolyticus*.

## Materials and Methods

### Animals, Ethics Statement, and Bacterial Strains

Healthy *S. paramamosain* (bodyweight 69.07 ± 23.99 g) were purchased from the Tulou Putou farm (Zhangpu, Zhangzhou, Fujian Province, China) in June 2018. Crabs were maintained in a self-built recycling cultivation system (seawater temperature at 26°C, salinity at 18‰–20‰, and fed with clams or oysters once a day at night) for 10 days to acclimatize to their new environment before experimentation. In all subsequent experiments, ≥6 individuals were sampled at different times. All of the study design and animal experiments were conducted following the guidelines of Fujian Agriculture and Forestry University’s Animal Care and Use Committee.

*V. parahaemolyticus* (isolated from diseased mud crab and preserved in our laboratory) was cultured in LB media (containing 10 g/L peptone, 5 g/L yeast extract, and 10 g/L NaCl) at 37°C for 24 h and harvested *via* centrifugation (6000 × *g*, 10 min, 4°C). The pellet was washed and resuspended in sterilized physiologic (0.9%) saline for crabs (PS), which comprises (in g/L), NaCl (26.00), KCl (0.85), CaCl_2_ (1.50), MgCl_2_ (2.33), H_3_BO_3_ (0.55), NaOH (0.02), and Na_2_SO_4_ (3.00). The suspension was adjusted to a final concentration of 3.1×10^7^ CFU/mL and termed the “live *V*. *parahaemolyticus* suspension.” Heat-killed *V*. *parahaemolyticus* was acquired by autoclaving the suspension for 30 min at 60°C. Subsequently, the plate coating was used to determine whether all the bacteria had been killed.

### Bacterial Challenge

**Figure 1:**
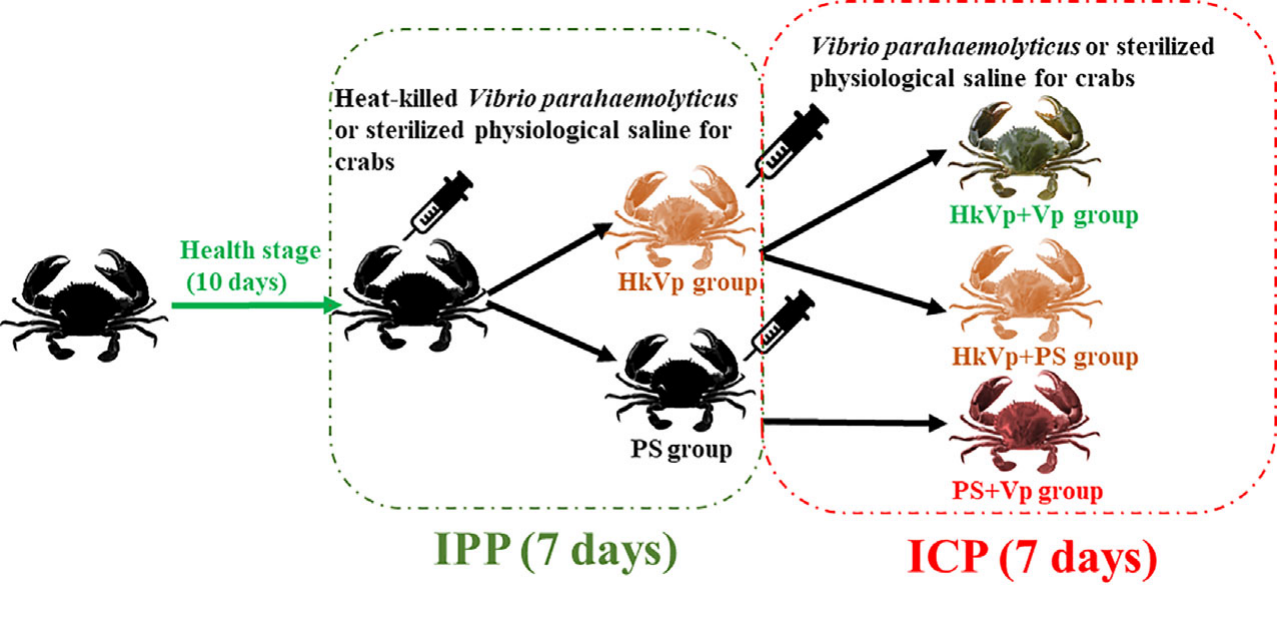
Experimental design of *S. paramamosain* receiving heat-killed *V. parahaemolyticus* (IPP) and live *V. parahaemolyticus* (ICP).

The experiment was split into the immune priming phase (IPP) and immune challenge phase (ICP). For the IPP, 250 crabs received an injection of PS (100 μL) and were employed as the PS group. Another 250 crabs received the heat-killed *V*. *parahaemolyticus* suspension at an effective CFU per gram of crab of 10^4^ and were employed as the HkVp group. At 6, 9, and 72 h of the IPP, 36 crabs from the PS grup and HkVp groups had their hemolymph and hepatopancreas collected, respectively (*N* = 6). After 168 h of the IPP, in the ICP, all the remaining crabs from the PS group received an injection of live *V*. *parahemolyticus* (5.0×10^3^ CFU/g) and were employed as the PS+Vp group. Meanwhile, the remaining crabs from the HkVp group were again divided into the HkVp+Vp group (received an injection of live *V*. *parahaemolyticus* with the effective CFU per gram of crab of 5.0×10^3^) and HkVp+PS group (received an injection of 100 μL of PS). All the concentrations of *V*. *parahaemolyticus* mentioned above were determined by the mortality of mud crabs after injection of *V. parahaemolyticus* in the pre-experimental stage. Six crabs from each group had hemolymph and hepatopancreas collected at 6, 9, and 72 h of the ICP. The experimental design is illustrated in **Figure 1**.

### Survival Rate

The survival rate of crabs was measured at different time points after challenge with live *V*. *parahaemolyticus* (5.0×10^3^ CFU/g) after crabs had been pretreated with heat-killed *V*. *parahaemolyticus*. Survival was followed daily, and dead crabs were removed from tanks. Survival rates were analyzed for statistical differences between treatments by log-rank test on Kaplan-Meier survival curves using the computer software package GraphPad Prism v. 7.0.

### Isolation of Total RNA and Reverse Transcription

Hepatopancreas samples were stored immediately in liquid nitrogen and RNAlater, respectively, until used for RNA isolation. Hemocytes were isolated rapidly by centrifugation at 2000 × g for 10 min at 4°C from the hemolymph. The latter was harvested from the last walking leg with an equal volume of ice-cold anticoagulant buffer (contains 0.45 M NaCL, 0.1 M glucose, 30 mM trisodium citrate, 26 mM citric acid, and 10 mM EDTA) and frozen immediately in liquid nitrogen until used for RNA isolation.

Total RNA was extracted from different tissues of unchallenged and challenged samples using TRIzol^®^ Reagent (Invitrogen, Carlsbad, CA, USA) according to manufacturer protocols. The quality of total RNA was assessed by agarose gel electrophoresis and quantified by spectrophotometry using a NanoDrop™ 1000 (Thermo Fisher, Waltham, MA, USA). Then, 2 µg of total RNA and 2 µL of random primers (10 µM) were used to synthesize cDNA by M-MLV reverse transcriptase (Invitrogen, Carlsbad, CA, USA). Then, synthesized complementary (c)DNA was diluted 10-fold and 100-fold and stored at −20°C until use.

### Quantitative Real-Time Polymerase Chain Reaction (qRT-PCR) and Statistical Analyses

Fourteen pairs of gene-specific primers for the genes of members of the TLR signaling pathway and AMPs (**Additional File 1: Table S1**) were designed to amplify products of 150–250 bp from cDNA, respectively. To compare the relative expression of these 14 genes in the samples, the housekeeping gene *EF1a* (GenBank ID: JQ824130) was also amplified with the same cDNA samples (**Additional File 1: Table S1**). The PCR was carried out in a LightCycler™ 480 Real-time Thermal Cycler (Roche, Basel, Switzerland) following manufacturer instructions with a total reaction volume of 20 µL [10 µL of 10× SYBR Green Master Mix (Yeasen Biotechnology, Shanghai, China), 9 µL of original cDNA (1:100 dilution), and 0.5 µL of each primer (10 µM)]. The thermal profile for RT-PCR was 1 min at 95°C, followed by 40 cycles at 95°C for 15 s and 60°C for 1 min. Melting curves were also plotted (60°C–90°C) to ensure that a single PCR product was amplified for each pair of primers. The comparative CT method (ΔCT = CT of target gene − CT of *EF1a* and ΔΔCT = ΔCT of any sample – the calibrator sample) was used to calculate the relative expression of all 14 genes (51). Then, the data were subjected to a Student’s *t*-test to determine the difference in the mean values among the treatments, and the significant difference in expression was shown at *P* < 0.05.

Violin plots were created using OmicShare tools, a free online platform for data analyses (www.omicshare.com/tools), to measure the expression of the genes related to the TLR signaling pathway and AMPs in each tissue at different time points under dissimilar conditions. Heat maps were created using R Programming Language v3.5.3 (R Center for Statistical Computing, Vienna, Austria) to visualize gene-expression data graphically. The intermolecular interaction network of TLR signaling pathway-related genes was constructed using String v11.0 (https://string-db.org/) and Cytoscape 3.7.1 (https://cytoscape.org/).

## Results

### Increased Survival Rate of *S. paramamosain* After the ICP With Live *V. parahaemolyticus*

**Figure 2:**
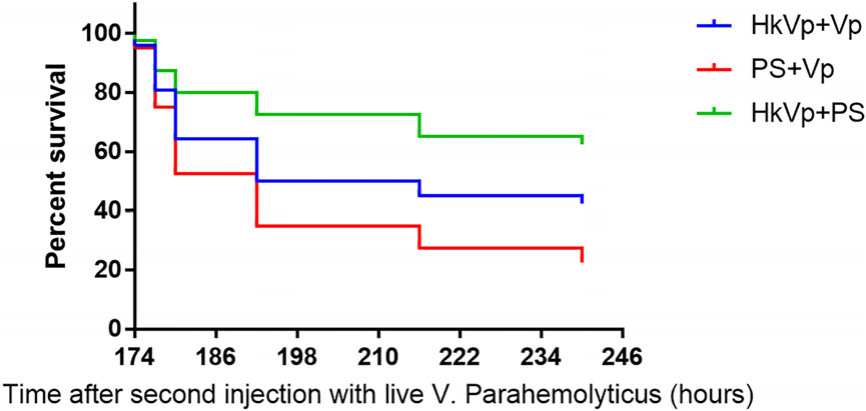
Cumulative survival using the Kaplan-Meier method following the ICP with live *V. parahaemolyticus* after previous IPP with heat-killed *V. parahaemolyticus* (HkVp group) or physiologic (0.9%) saline (PS group) in *S. paramamosain*. “168–240 h” denotes the time started after the ICP. This survival experiment was repeated three times.

To understand the immune effect of *V. parahaemolyticus* during the IPP and ICP, we counted the number of *S. paramamosain* that survived. After the IPP, the mortality of the PS group was not significantly different to that of the HkVp group. At 7 days after the IPP, animals entered the ICP with a lethal dose of live *V. parahemolyticus*. There was a difference in the survival rate between the HkVp+Vp group and PS+Vp group from 9 h, and it became greater and more significant with increasing time, and the log-rank test shows a significant difference in survival rate between HkVp+Vp group and the other groups after the ICH (*p* < 0.05) (**Figure 2**). The contrast in the survival rate between the HkVp+Vp and PS+Vp groups indicates that the immune protection of *S. paramamosain* was enhanced when they were infected again by *V. parahaemolyticus* (**Figure 2**).

### Changes in mRNA Expression of Genes Involved in the TLR Signaling Pathway

In hemocytes, mRNA expression of *MyD88* in the HkVp+Vp and PS+Vp groups was upregulated 6 h after the ICP, and it peaked significantly at 72 h in the PS+Vp group compared with that in the HkVp+Vp and HkVp+PS groups (*p* < 0.05). After the IPP, the expression of *Dorsal* in the HkVp group was upregulated at first and peaked significantly at 72 h (*p* < 0.05). After the ICP, the HkVp+Vp and PS+Vp groups seemed to adopt two immune modes. *Dorsal* expression in the HkVp+Vp group showed a downregulation trend that was significantly lower than that in the HkVp+PS group at 9 and 72 h, whereas mRNA expression in the PS+Vp group peaked at 72 h and was significantly more upregulated than that in the HkVp+PS and HkVp+Vp groups (*p* < 0.05). Similar to the expression pattern of *Dorsal*, we found that the expression of *TLR* and *Pelle* also showed a downregulation trend after the ICP (*p* < 0.05). Conversely, the expression of *Spaetzle* and *Cactus* showed a significantly upregulated pattern at almost all time points after the ICP (*p* < 0.05). Expression of *Dscam* in the HkVp+Vp group peaked 9 h after the ICP and was upregulated significantly compared with that in the HkVp+PS and PS+Vp groups. The significant difference was still present at 72 h (*p* < 0.05) (**Figure S1**).

In the hepatopancreas, mRNA expression of *MyD88* in the HkVp group was upregulated significantly 9 h after the IPP (*p* < 0.05) and showed a significant downregulation trend at 6 and 9 h in the HkVp+Vp group (*p* < 0.05). Expression of *Dscam* in the HkVp+Vp group peaked 9 h after the ICP and was upregulated considerably more than that in the HkVp+PS and PS+Vp groups (*p* < 0.05). The expression of *TLR* and *Dorsal* in the HkVp+Vp group was significantly weaker than that in the PS+Vp group (*p* < 0.05). After the IPP, *Pelle* expression in the HkVp group was significantly upregulated at 6 and 72 h (*p* < 0.05), whereas it was also upregulated significantly 6 and 9 h after the ICP in the HkVp+Vp group. Analogous to the expression pattern of *Pelle*, the expression of *Spaetzle* and *Cactus* also showed a considerably upregulated trend at different time points after the ICP (*p* < 0.05) (**Figure S2**).

### Changes of mRNA Expression of AMPs Genes

In hemocytes, mRNA expression of *Arasin* was upregulated significantly at 6 h in the HkVp+Vp group and at 72 h in the PS+Vp group after the ICP. Compared with the HkVp+PS and PS+Vp groups, expression of *Crustin*, *ALF3*, and *ALF4* in the HkVp+Vp group after the ICP peaked at 72, 6, and 9 h, respectively. The expression pattern of *ALF6* was identical to that of *ALF3* and was upregulated significantly 6 h after the ICP in the HkVp+Vp group (*p* < 0.05). *ALF5* maintained a high level of expression at all time points after the ICP in the HkVp+Vp group compared with that in the HkVp+PS and PS+Vp groups (*p* < 0.05). Slightly different from the expression pattern of *ALF5*, *Hyastatin* showed significantly high expression 6 and 9 h after the ICP in the HkVp+Vp group (*p* < 0.05) (**Figure S3**).

In the hepatopancreas, mRNA expression of *Arasin* was maintained at a significantly high level at all time points after the ICP in the HkVp+Vp group (*p* < 0.05). *ALF3*, *ALF5*, and *ALF6* also had the same pattern of expression as that of *Arasin*. *Crustin* expression in the HkVp+Vp group was upregulated significantly 9 and 72 h after the ICP than that in the HkVp+PS and PS+Vp groups (*p* < 0.05). Nine hours after the ICP, *ALF4* and *Hyastatin* in the HkVp+Vp group showed significantly high expression. However, *ALF4* expression at 6 and 72 h was much lower than that in the PS+Vp group, and *Hyastatin* expression was also upregulated significantly 9 and 72 h after the IPP (*p* < 0.05) (**Figure S4**).

### Visualization of the Expression Data of Genes of the TLR Signaling Pathway and AMPs

**Figure 3:**
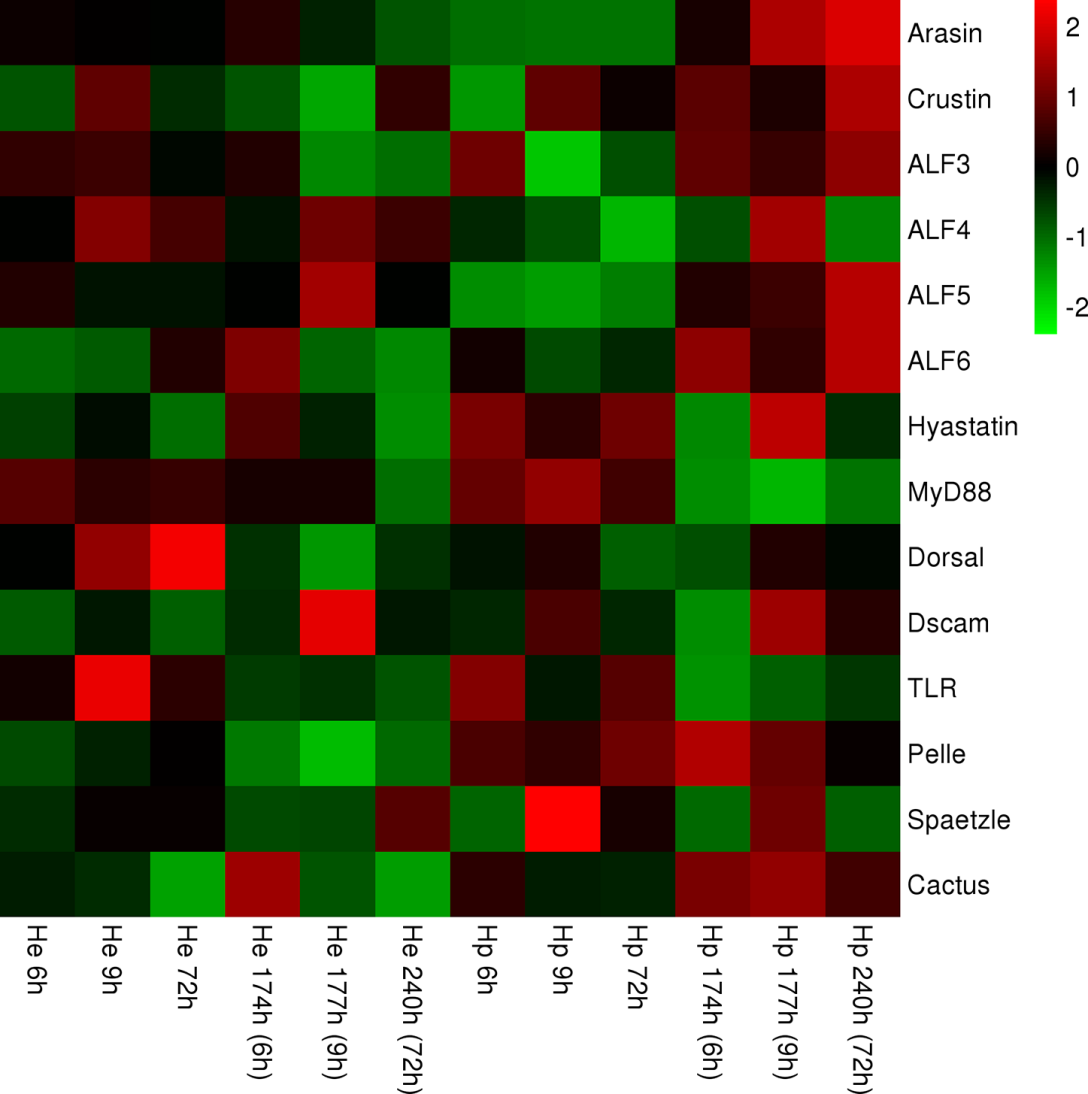
Heat map representing expression of genes of members of the TLR signaling pathway and AMPs in hemocytes and the hepatopancreas, respectively, containing continuous time points [IPP (6, 9, and 72 h) and ICP (174, 177, and 240 h)]. The color scale at the far right of the heat map represents expression, whereby red, green, and black indicate upregulated, downregulated, and unaltered expression, respectively.

After standardizing all the data, heat maps exhibit the different expression of genes of the TLR pathway or AMPs in the hepatopancreas and hemocytes after injection at different time points. Some of the same genes in the two tissues show different expression patterns. This result suggests that different regulatory modes of expression of genes in the TLR signaling pathway and AMP genes were activated in the hepatopancreas and hemocytes in the IPP and ICP (**Figure 3**).

**Figure 4:**
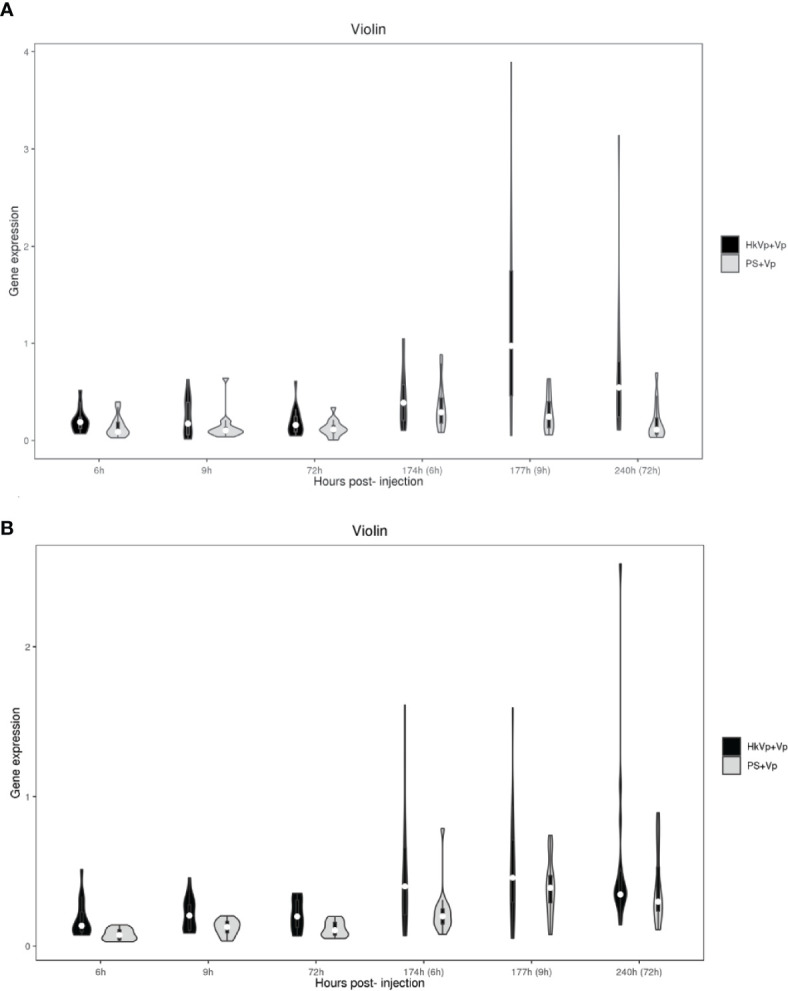
Violin plots representing mRNA expression of genes of members of the TLR signaling pathway and AMPs. **(A)** mRNA expression of genes in the hepatopancreas in the HkVp+Vp group compared with that in the PS+Vp group. **(B)** mRNA expression of genes in hemocytes in the HkVp+Vp group compared with that in the PS+Vp group. The *x*-axis denotes the hours after bacterial injection [IPP (6, 9, and 72 h) and the ICP (174, 177, and 240 h)]. The *y*-axis represents expression of genes of members of the TLR signaling pathway and AMPs.

Violin plots are used to summarize the differences in gene expression in hemocytes and the hepatopancreas, respectively. All genes of the TLR signaling pathway or AMPs at different time points (6, 9, 72, 174, 177, and 240 h) show that the different expression (i) was higher in hemocytes than that in hepatopancreas, (ii) became more significant in the hepatopancreas from 9 h in the ICP, (iii) became more significant in hemocytes at all time points of the ICP, and (iv) was greater at 9 h of the ICP in the hepatopancreas and hemocytes (**Figures 4A, B**).

### The Molecular Network of the TLR Signaling Pathway Constructed by Cytoscape

**Figure 5:**
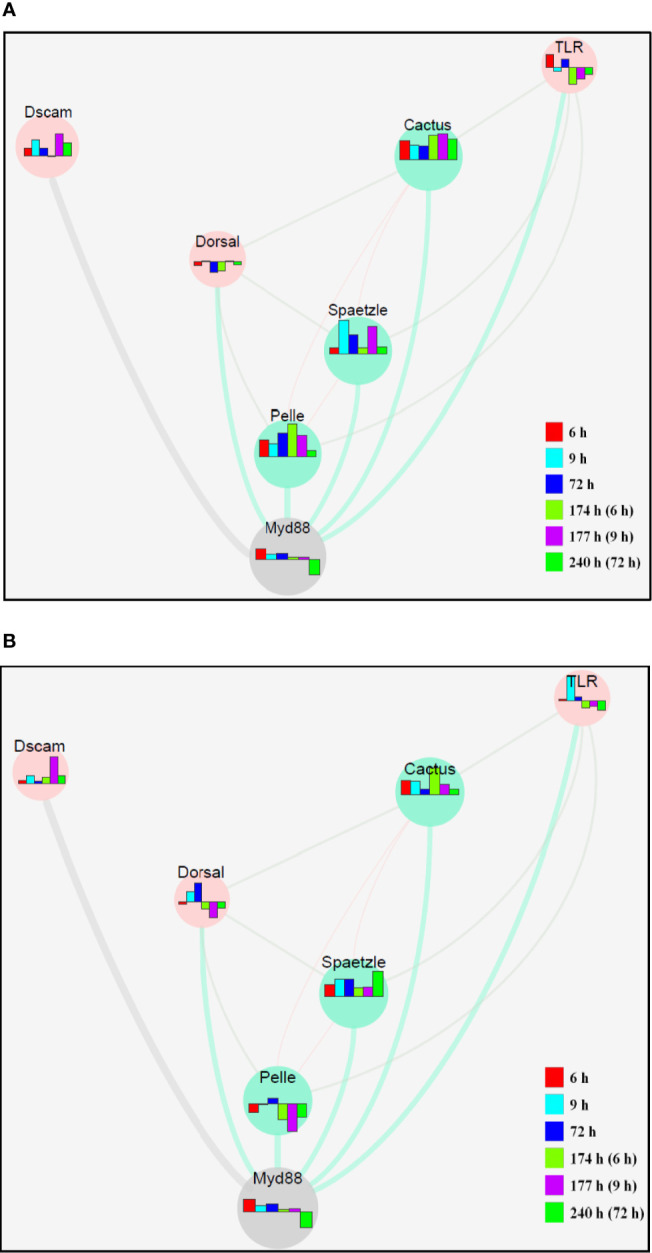
The molecular network of related genes in the TLR signaling pathway in response to secondary challenge by *V. parahaemolyticus* in the hepatopancreas **(A)** and hemocytes **(B)** of *S. paramamosain* constructed by Cytoscape. The bar chart represents the different expression patterns of each gene, and the different colors of the bar chart represent different time phases.

The molecular network of the related genes in the TLR signaling pathway created using Cytoscape indicates gene interactions at a discrete time point after injection (**Figure 5**). In terms of expression of different genes, there are considerable differences between the hepatopancreas (**Figure 5A**) and hemocytes (**Figure 5B**) according to this interaction network.

## Discussion

In mammals, health maintenance and disease prevention are promoted owing to studies of enhanced immune protection and their underlying immune mechanisms. Based on numerous studies in vertebrates, researchers are driven to explore the origin, evolution, and underlying mechanism of enhanced immune protection in invertebrates. Nevertheless, exploration of its evolution is limited (52).

Early studies on adaptive immunity focus mainly on the identification of lymphocytes and their receptors as well as molecules in the major histocompatibility complex. However, since the discovery of alternative mechanisms of adaptive immunity based on variable lymphocyte receptor in jawless vertebrates, Ig-dependent adaptive immunity ceased to be regarded as the only solution to enhance immune protection (53). In recent years, increasing attention has been paid to the long-neglected role of immune protection in invertebrates.

It is reported that immune protection after identical stimulation is significantly stronger in invertebrates than that in an unvaccinated group and that the effect is similar to that of a vaccine (3, 37, 38, 54–56). A mechanism of enhanced immune protection is demonstrated, but it is also reported to be associated with several factors: the interaction between different hosts and pathogens, the induced dose, and the duration (57).

Initially, crabs were immune-primed (IPP) with heat-killed *V. parahaemolyticus*. Then, they were immune challenged (ICP) with live *V. parahaemolyticus* with a lethal dose 7 days after the IPP. The survival rate of crabs after the ICP was the most direct evidence to judge the existence of immune priming in crabs. The difference in the survival rate between the HkVp+Vp and PS+Vp groups began to appear 9 h after the ICP, and it built up gradually over time. At 72 h, the difference between the two groups reached 20%. This result suggests (i) enhanced immune protection was initiated after the primary exposure to the same strain of *V. parahaemolyticus* and (ii) the existence of immune priming in *S. paramamosain*. Our finding of an enhanced immune response is in agreement with data from other studies showing a higher immune response could be induced by stimulation with inactivated pathogens in shrimps and crabs (39, 58). In other invertebrates, the upregulation of the immune response after repeated infection by the same pathogen is widespread, and it is considered to denote immune priming (56, 59, 60).

AMPs are vital components of the host innate immune response against microbial invasion (46). Pathogen infection can activate the TLR signaling pathway and its downstream signaling molecules; then the signal is translocated to the nucleus to induce the release of AMPs. AMPs, such as ALF3–6, Crustin, Arasin, and Hyastatin, are reported to be effectors of the immune response against infection by Gram-positive and Gram-negative bacteria in *S. paramamosain*. In the present study, expression of these AMP genes was also measured to ascertain if they were involved in the immune priming of *S. paramamosain*. We note significantly high expression of *Arasin*, *ALF3*, *ALF4*, *ALF6*, and *Hyastatin* in the hemocytes of the HkVp+Vp group in the initial stage after ICP. Simultaneously, *Crustin* expression peaked 72 h after the ICP. In the hepatopancreas of crabs in the HkVp+Vp group, the expression patterns of *ALF4* and *Hyastatin* were similar to those in hemocytes.

Interestingly, expression of *Arasin*, *Crustin*, *ALF3*, and *ALF6* was activated at the initial stage after the ICP and was upregulated significantly throughout the period. Moreover, *ALF5* expression was upregulated markedly throughout the ICP in hemocytes and in the hepatopancreas. Taken together, these results suggest that AMP genes participate in the immune priming of *S. paramamosain*, but in different tissues, the regulation mode is not identical.

The TLR signaling pathway is vital for the immune system of organisms due to its regulatory effect upon bacterial infection: It positively regulates the expression of downstream innate immune-related genes (48, 61). In the TLR signaling pathway of *Drosophila*, a platform is provided for Pelle to permit incorporation and form a trimeric complex by MyD88 recruiting its upstream-activated TLR and downstream cytosolic adaptor Tube. Meanwhile, the complex can also allow further activation of Pelle and then induce activation of Dorsal expression (62). Studies of various bacterial-induced Dscam isoforms in arthropods have focused on assays of gene silencing, bacterial binding assays, and pathogen clearance (29, 63, 64). The speed and intensity of the response of Dscam to pathogen infection after a previous encounter suggest specific immune priming (65, 66). Several studies have centered on Dscam and its involvement in immune priming in invertebrates, but those studies are on only a few species (31, 64, 67, 68).

We measured expression of seven genes associated with the TLR signaling pathway by qRT-PCR after pre-exposure of *V. parahaemolyticus* to evaluate whether these genes are involved in enhanced immune protection. Unlike the expression pattern of AMP genes, which were upregulated in the hepatopancreas and hemocytes, the expression pattern of genes in the TLR signaling pathway was quite different after the ICP. In hemocytes of the HkVp+Vp group, *Spaetzle* was activated in the early stage after *V. parahaemolyticus* infection and showed high expression throughout the ICP. *MyD88* had high transient expression 6 h after infection and then showed a stable expression pattern. However, expression of genes, such as *TLR*, *Pelle*, and *Dorsal*, was inhibited after the ICP, especially in the advanced stage of infection. *Cactus* was activated at the early stage after infection, and in the later stage, the effect was weakened. *Dscam* expression reached its peak at 9 h. In the hemocytes of the PS+Vp group, *Spaetzle* was activated in the early stage after infection, whereas *TLR* and *Pelle* were activated in the later stage. *MyD88* and *Dorsal* were activated through the ICP, and expression of *Cactus* and *Dscam* was relatively unchanging. In the hepatopancreas, the expression and regulation patterns of the genes stated above were different from those in hemocytes. Similar to results in *Drosophila*, in studies of *S. paramamosain*, TLR, Pelle, and MyD88-mediated antibacterial models are reported, and in those models, toll-associated downstream signaling molecules (such as Spaetzle, TLR, Cactus, and Dorsal) participated in the immune response to Gram-negative bacteria (50, 69). Also, studies have shown that at least 36,736 isoforms of Dscam could potentially be generated in *S. paramamosain* and that the pathogen-induced Dscam isoforms could enhance self-protection upon pathogen infection (70).

Interestingly, the expression patterns of most genes in the PS+Vp group after the ICP were similar to those reported previously, which were upregulated significantly after infection by Gram-negative bacteria. Hence, the classical TLR signaling pathway was the main way to resist infection by *V. parahaemolyticus* in the PS+Vp group. However, after pre-exposure to *V. parahaemolyticus*, expression of these genes in the HkVp+Vp group suggests that, whether in hemocytes or the hepatopancreas, the classic TLR signaling pathway could not be activated by *V. parahaemolyticus* (or other compensatory mechanisms were activated simultaneously), which led to inhibition of expression of TLR, Pelle, and Dorsal after the ICP. In addition, irrespective of which type of pathway is activated, Spaetzle played a major part in this process. More importantly, expression of most genes of the TLR pathway had long-lasting changes after the ICP. This observation suggests that the TLR pathway has the most important role in enhanced immune protection against reinfection by *V. parahaemolyticus* and is involved in the process of immune priming of *S. paramamosain*. In addition, except for *Dscam*, there were complex interactions among the other six genes, which is worthy of more in-depth research in the future.

## Conclusions

The present study provides evidence of the immune priming of *S. paramamosain* to *V. parahaemolyticus*. The differences between hemocytes and the hepatopancreas between the HkVp+Vp and the PS+Vp groups were studied by macroscopic survival rate and molecular-biology methods.

Our study elicits two main conclusions. First, our experiments indicate that immune priming is present in *S. paramamosain* when it was infected repeatedly by *V. parahaemolyticus* and that the related genes, which may have been involved in the immune priming of hemocytes and hepatopancreas of *S. paramamosain*, were identified. Second, the TLR signaling pathway, but not the classical style, was activated to participate in the immune priming of *S. paramamosain*. Our results provid further molecular insight into enhanced immune protection in *S. paramamosain*. Our data is valuable for understanding the mechanisms of enhanced immune protection in invertebrates.

## Data Availability Statement

The datasets presented in this article are not readily available because: The data will be available upon request. Requests to access the datasets should be directed to zhangziping@hotmail.com.

## Author Contributions

YW and ZZ conceived the study and designed the experiments. XZ conducted the experiments and wrote the manuscript. XYZ analyzed the data. YS conducted the experiments. YW and ZZ checked and modified the manuscript. All authors contributed to the article and approved the submitted version.

## Funding

The work was supported by the Natural Science Foundation of China (No. 31672681), the Discipline Development Grant from College of Animal Sciences FAFU (712018R0404), Open fund project of Fujian Engineering Research Center of Aquatic Breeding and Healthy Aquaculture (DF20902), and the Startup Fund of Fujian Agriculture and Forestry University (61201401201) for ZZ.

## Conflict of Interest

The authors declare that the research was conducted in the absence of any commercial or financial relationships that could be construed as a potential conflict of interest.

## Supplementary Material

Supplementary Figure 1 Expression of genes of members of the TLR signaling pathway in hemocytes after the IPP and ICP. The *x*-axis denotes hours after bacterial injection [IPP (6, 9, and 72 h) and the ICP (174, 177, and 240 h)]. The *y*-axis reflects expression of genes of members of the TLR signaling pathway. The significant difference between the challenged group and control group is indicated by * at *p* < 0.05. *EF1-α* served as a reference gene.

Supplementary Figure 2 Expression of genes of members of the TLR signaling pathway in the hepatopancreas after IPP and ICP. The *x*-axis denotes hours after bacterial injection [IPP (6, 9, and 72 h) and the ICP (174, 177, and 240 h)]. The *y*-axis reflects expression of genes of members of the TLR signaling pathway. The significant difference between the challenged group and control group is indicated by * at *p* < 0.05. *EF1-α* served as a reference gene.

Supplementary Figure 3 Expression of AMP genes in hemocytes after the IPP and ICP. The *x*-axis denotes hours after bacterial injection [IPP (6, 9, and 72 h) and the ICP (174, 177, and 240 h)]. The *y*-axis reflects expression of genes of members of the TLR signaling pathway. The significant difference between the challenged group and control group is indicated by * at *p* < 0.05. *EF1-α* served as a reference gene.

Supplementary Figure 4 Expression of AMP genes in the hepatopancreas after the IPP and ICP. The *x*- axis denotes hours after bacterial injection [IPP (6, 9, and 72 h) and the ICP (174, 177, and 240 h)]. The *y*-axis reflects expression of genes of members of the TLR signaling pathway. The significant difference between the challenged and control groups is indicated by * at *p* < 0.05. *EF1-α* served as a reference gene.
