# Supplementary figures and images for: Enhanced Immune Protection of Mud Crab Scylla paramamosain in Response to the Secondary Challenge by Vibrio parahaemolyticus
Source: Front Immunol. 2020 Oct 20;11:565958. doi: 10.3389/fimmu.2020.565958 (PMC7606287; doi:10.3389/fimmu.2020.565958)

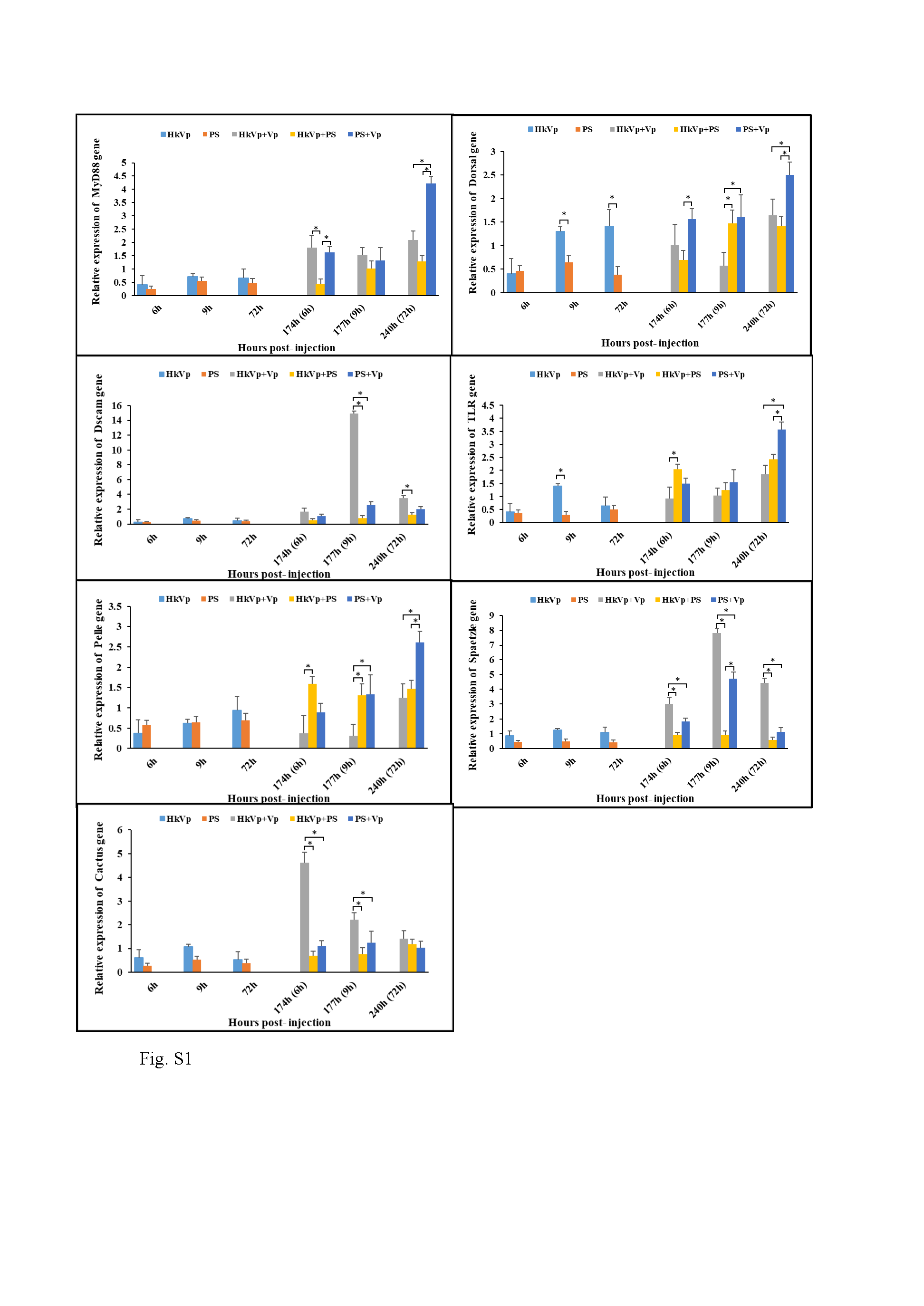

Supplement: Supplementary Figure 1 — Expression of genes of members of the TLR signaling pathway in hemocytes after the IPP and ICP. The x-axis denotes hours after bacterial injection [IPP (6, 9, and 72 h) and the ICP (174, 177, and 240 h)]. The y-axis reflects expression of genes of members of the TLR signaling pathway. The significant difference between the challenged group and control group is indicated by * at p < 0.05. EF1-α served as a reference gene. [file Image_1.tiff]

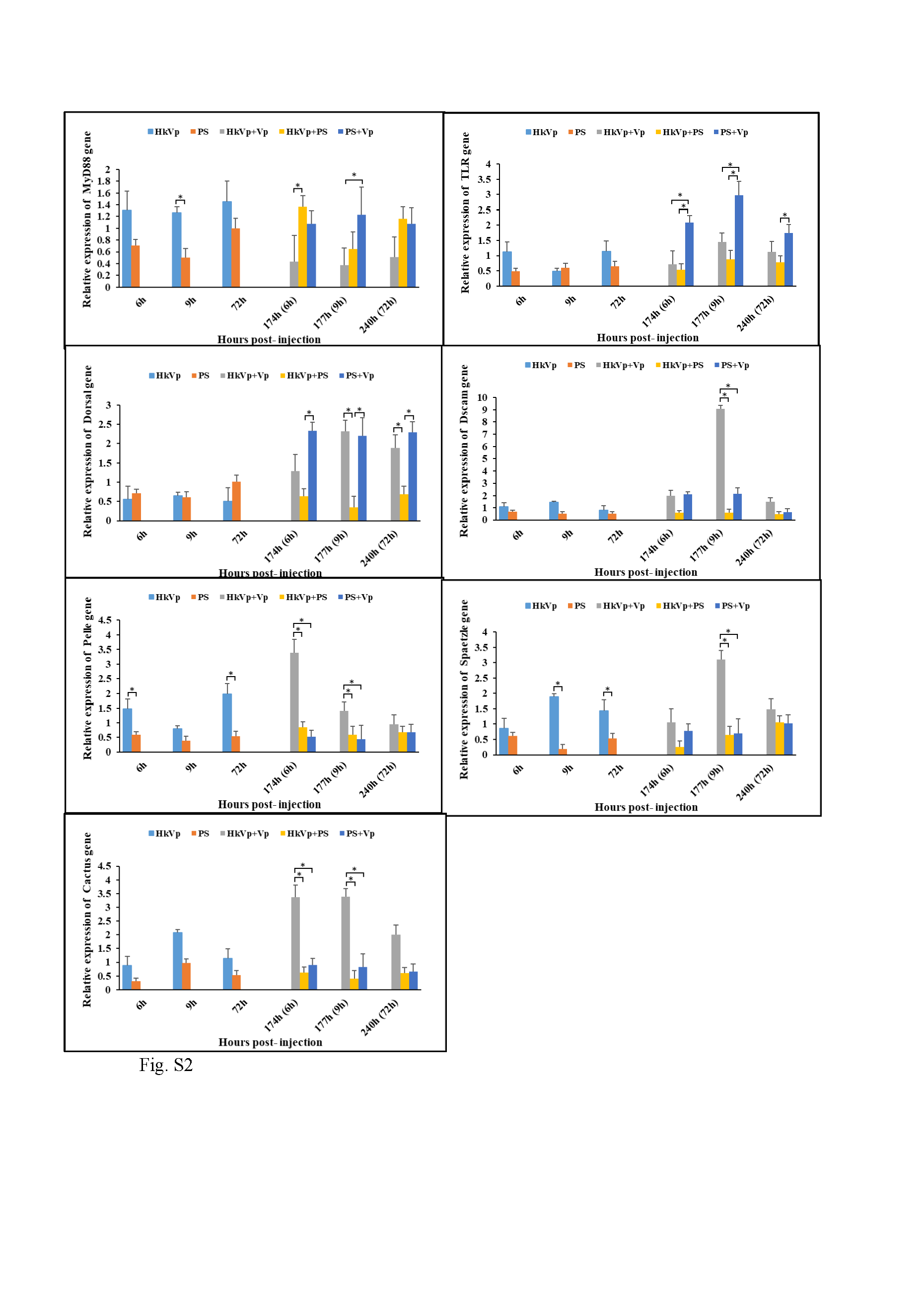

Supplement: Supplementary Figure 2 — Expression of genes of members of the TLR signaling pathway in the hepatopancreas after IPP and ICP. The x-axis denotes hours after bacterial injection [IPP (6, 9, and 72 h) and the ICP (174, 177, and 240 h)]. The y-axis reflects expression of genes of members of the TLR signaling pathway. The significant difference between the challenged group and control group is indicated by * at p < 0.05. EF1-α served as a reference gene. [file Image_2.tiff]

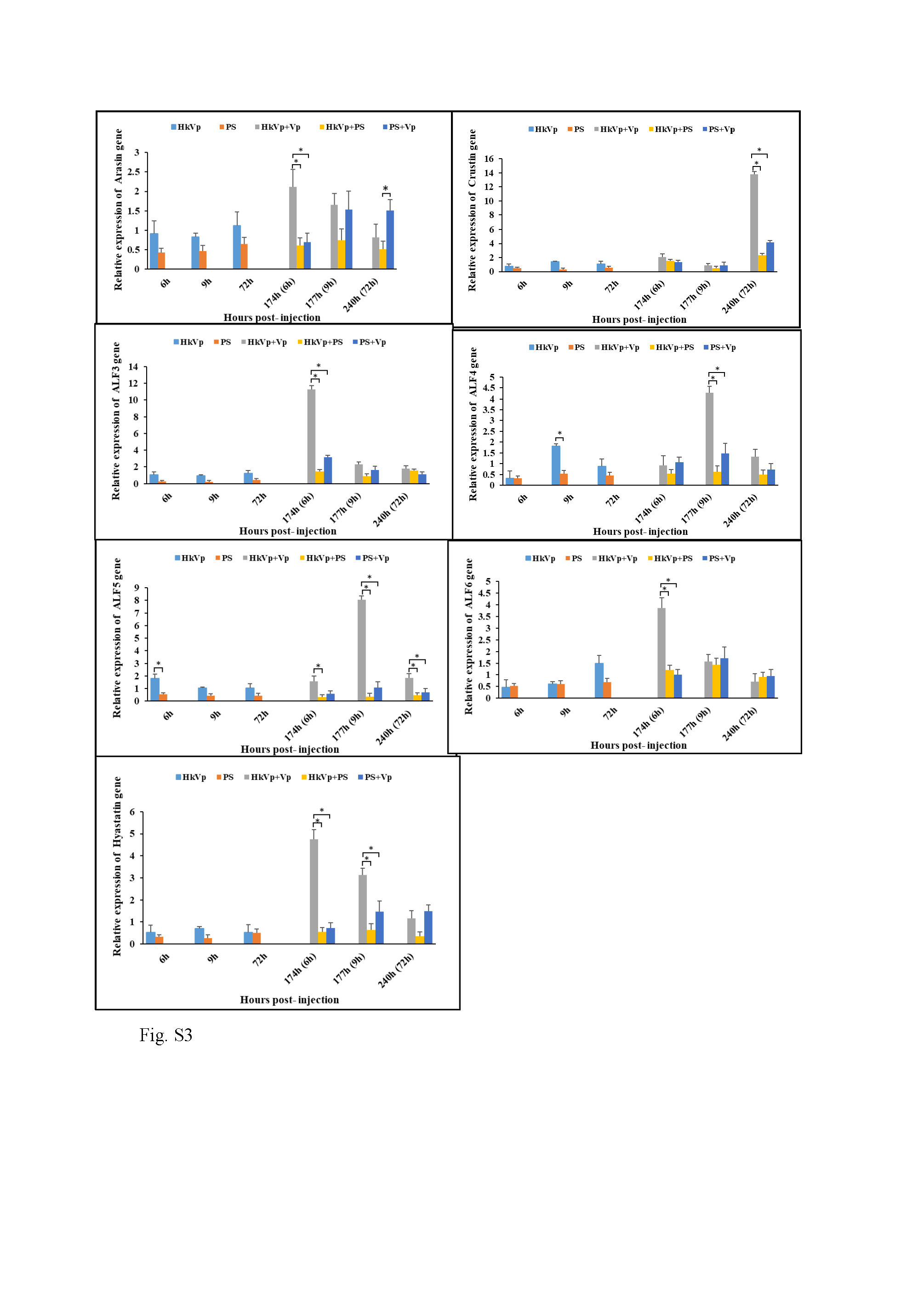

Supplement: Supplementary Figure 3 — Expression of AMP genes in hemocytes after the IPP and ICP. The x-axis denotes hours after bacterial injection [IPP (6, 9, and 72 h) and the ICP (174, 177, and 240 h)]. The y-axis reflects expression of genes of members of the TLR signaling pathway. The significant difference between the challenged group and control group is indicated by * at p < 0.05. EF1-α served as a reference gene. [file Image_3.tiff]

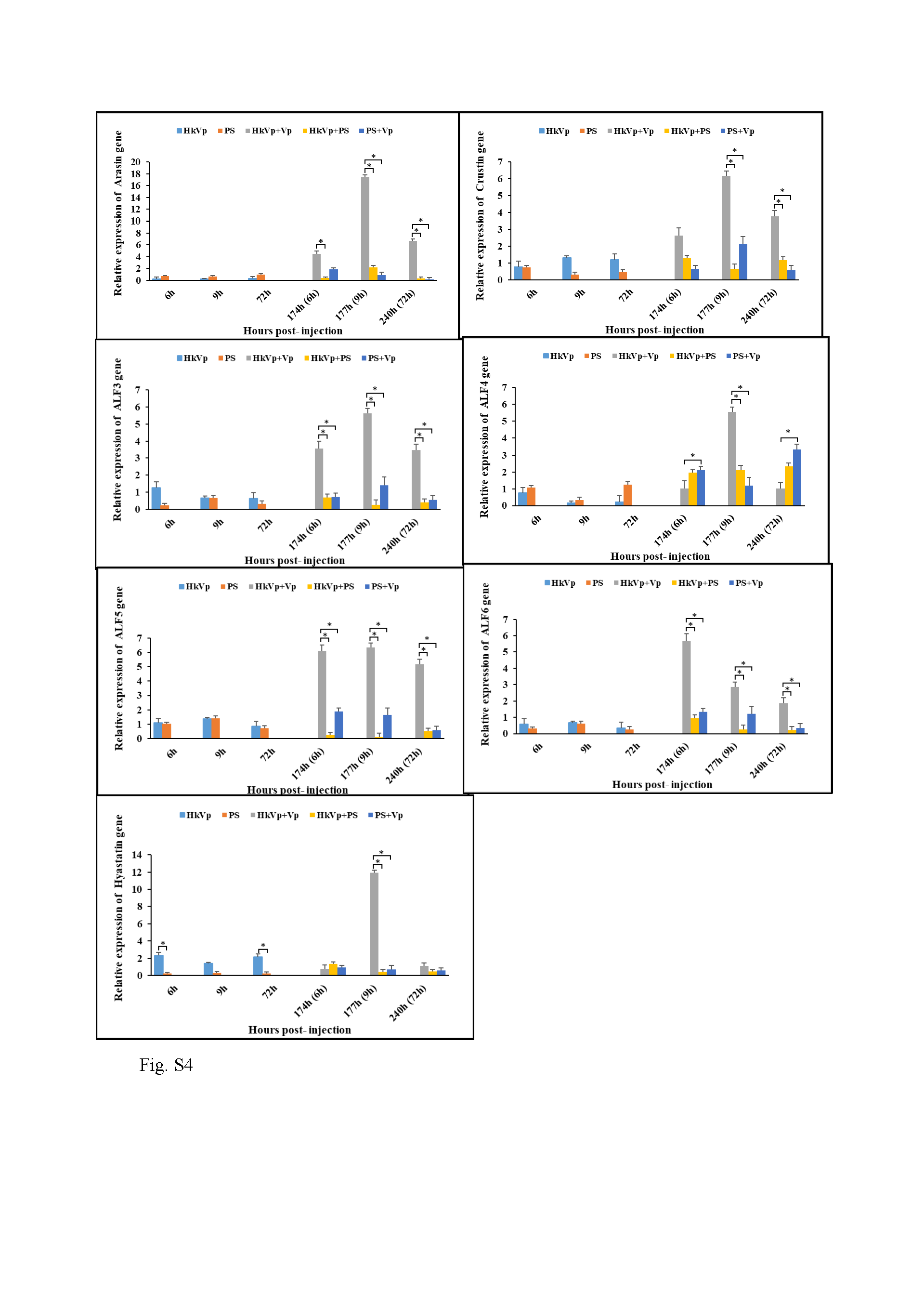

Supplement: Supplementary Figure 4 — Expression of AMP genes in the hepatopancreas after the IPP and ICP. The x- axis denotes hours after bacterial injection [IPP (6, 9, and 72 h) and the ICP (174, 177, and 240 h)]. The y-axis reflects expression of genes of members of the TLR signaling pathway. The significant difference between the challenged and control groups is indicated by * at p < 0.05. EF1-α served as a reference gene. [file Image_4.tiff]
