# Supplementary material for: Enhanced Immune Protection of Mud Crab Scylla paramamosain in Response to the Secondary Challenge by Vibrio parahaemolyticus
Source: Front Immunol. 2020 Oct 20;11:565958. doi: 10.3389/fimmu.2020.565958 (PMC7606287; doi:10.3389/fimmu.2020.565958)
Supplement: Supplementary file 5 [file Table_1.docx]

Additional file 1 Table S1.

Primers used in this article

| Primer name | Nucleotide sequence (5'→3') | Purpose |  |
| --- | --- | --- | --- |
| Dscam-F | GGGGGTACTATTGCACCGATTC | qRT-PCR | |
| Dscam-R | TACAGCTGAGACCACAGGGACG |  | |
| MyD88-F | AGAATACGGGAACTGTCTGGGT |  | |
| MyD88-R | TGGTGACTGTGATGGAGATGGT |  | |
| Dorsal-F | GCATCTATGGTTGATGCACACTTG |  | |
| Dorsal-R | GGCATGTAAGATGAAGATGCACTG |  | |
| TLR-F | AGCCAAAGAAGAAGCAGATAAGAAG |  | |
| TLR-R | AGTCTCGGTAGTGGAGGCACA |  | |
| Pelle-F | ACATCTGGATAACACCCGTCTC |  | |
| Pelle-R | GGCATGAACTGGTACACAAGG |  | |
| Spaetzle-F | CTGGGCAGAACACACAGGAGTCT |  | |
| Spaetzle-R | GTACGATGAGCGGATCAAGCAGC |  | |
| Cactus-F | CGATGGTGGCGCATTTAACGAGAC |  | |
| Cactus-R | TAGTTGGTCAGGTCAGCAGCAAGG |  | |
| Arasin-F | ACTTCGACATGGAGCGACGCAC |  | |
| Arasin-R | TTAAGTCAAAGAAGCAAGAGTCAAC |  | |
| Crustin-F | GTAAATGTCCTTCAGTCCGCTCT |  | |
| Crustin-R | CAACAGTCTTGCATACGTGGTG |  | |
| ALF3-F | GAACGGACTCATCACACAGCAG |  | |
| ALF3-R | CACTTCCTTGTTCTCTTCGCTC |  | |
| ALF4-F | CACTACTGTGTCCTGAGCCGC |  | |
| ALF4-R | GTCCTCGCCTTACAATCTTCTG |  | |
| ALF5-F | CTTGAAGGGACGAGGTGATGAG |  | |
| ALF5-R | TGACCAGCCCATTCGCTACAG |  | |
| ALF6-F | ACAGGGCTATCGCAGACTTCG |  | |
| ALF6-R | GCACCTCTTTGGCACACTATTTG |  | |
| Hyastatin-F | TGACAACTGACCGCAGACGAC |  | |
| Hyastatin-R | GACAGGAAACATGACACCTGCAC |  | |
| EF1a -F | GGTGCTGGACAAGCTGAAGGC |  | |
| EF1a -R | CGTTCCGGTGATCATGTTCTTGATG |  | |
